# Changes in sleep duration and risk of metabolic syndrome: the Kailuan prospective study

**DOI:** 10.1038/srep36861

**Published:** 2016-11-18

**Authors:** Qiaofeng Song, Xiaoxue Liu, Wenhua Zhou, Xizhu Wang, Shouling Wu

**Affiliations:** 1 Department of Cardiology, Tangshan People’s Hospital, North China University of Science and Technology, Tangshan, China; 2 Department of Cardiology, Kailuan Hospital, North China University of Science and Technology, Tangshan, China

## Abstract

Using a large longitudinal data set spanning 4 years, we examined whether a change in self-reported sleep duration is associated with metabolic syndrome (MetS). Current analysis included 15,753 participants who were free of MetS during both 2006–2007 and 2010–2011. Sleep duration was categorized into seven groups: ≤5.5 h, 6.0–6.5 h, 7.0 h, 7.5–8.0 h, ≥8.5 h, decrease ≥2 h, and increase ≥2 h. Cox proportional hazards models were used to calculate hazard ratios (HRs) and their confidence intervals (CI) for MetS, according to sleep duration. Compared to the reference group of persistent 7-h sleepers, a decrease of ≥2 h sleep per night was associated with a higher risk of incident MetS (HR = 1.23, 95% CI = 1.05–1.44) in analyses adjusted for age, sex, sleep duration at baseline, marital status, monthly income per family member, education level, smoking status, drinking status, physical activity, body mass index, snoring status and resting heart rate. An increased risk of MetS incidence was also observed in persistent short sleepers (average ≤5.5 h/night; HR = 1.22, 95% CI = 1.01–1.50). This study suggests individuals whose sleep duration decreases ≥2 h per night are at an increased risk of MetS.

Metabolic syndrome (MetS) is closely linked to lifestyle and clustering of risk factors, including abdominal adiposity, hypertension, hypertriglyceridemia, low high-density lipoprotein (HDL) cholesterol and hyperglycemia. Given that MetS is becoming a pandemic, with prevalence rates between 20 and 30% among the adult population^1^, identification of modifiable risk factors associated with the development of MetS is important to public health. Prior cross-sectional studies suggest short sleep duration is significantly associated with increased risk of MetS^2^,^3^,^4^,^5^. Recent prospective analyses have also provided robust evidence of a similar association between short sleep duration and a higher incidence of MetS^6^,^7^,^8^. Conversely, other studies have shown an association between long sleep duration and MetS^9^,^10^. Thus, it is necessary to assess the temporal relationship between sleep duration and MetS.

Increasing concerns about reductions in night time sleep duration caused by our new 24/7 society^11^,^12^ cannot be addressed by studies based on a one-off measurement of sleep duration. Thus, it is impossible to determine from such studies^6^,^7^,^8^ whether associations observed between short or long sleep duration and MetS are generated by persistent exposure, or whether decreases or increases from a normal average of 7 h per night also confer risk. A significant decrease or increase in sleep duration has also been associated with increased all-cause mortality^13^. Cross-sectional data from the 2005–2006 National Health and Nutrition Examination Survey looked at the change in objective sleep duration and individual cardiometabolic risk factors^14^. Data from the Whitehall II study suggests that individuals whose sleep duration increases are at an increased risk of type 2 diabetes, although we are unaware of any population level investigations of change in sleep duration and incidence of MetS^15^.

In this study, we examined potential longitudinal associations between changes in sleep duration over a 4-year exposure period (the reference group of persistent 7-h sleepers ref. 16) and incidence of MetS in the subsequent 4-year period. Using a large prospective study from Kailuan, we took account of sleep duration and snoring status at baseline examination, as a potential confounder and mediator of these associations.

## Results

Table 1 shows the general characteristics of study participants according to the incidence of MetS. Baseline SBP, DBP, TC, BMI, TG, FBG, and RHR were significantly higher, and HDL-C was significantly lower in individuals who developed MetS compared with non-MetS (p < 0.001).

Table 2 shows baseline characteristics according to sleep duration. Significant association was found between sleep duration and age, sex, education level, income level, smoking status, drinking status, physical activity, BMI, SBP, DBP, FBG, TG, HDL-C, RHR, snoring status, history of stroke, myocardial infarction, and cancer (p < 0.001).

During an average 3.4-year follow-up, 6,302 participants (40.0%) developed MetS. Age, sex, sleep duration at baseline, sex, marital status, monthly income per family member, education level, smoking status, drinking status, physical activity, body mass index, snoring status and resting heart rate were designated as confounding factors in Model 2. After adjusting for these confounding factors, participants who slept ≤5.5 h per night had an increased risk of developing MetS compared with participants who persistently slept 7 h (HR = 1.22, 95% CI = 1.01–1.50; all p-values <0.05). Compared with the reference group of persistent 7-h sleepers, a decrease of ≥2 h sleep per night was associated with a higher risk of MetS incidence (HR = 1.23, 95% CI = 1.05–1.44; all p-values <0.05) (Table 3). Moreover, the association between decreased sleep duration and risk of MetS remained significant upon repeating our analysis and excluding individuals with stroke, myocardial infarction and cancer, respectively (Table 4).

## Discussion

Our findings suggest persistent short sleep (average <5.5 h) increases the risk of MetS incidence. In contrast, increased sleep duration was not associated with higher incidence of MetS compared with individuals persistently sleeping 7 h per night. Compared to these individuals, we also found that a decrease of ≥2 h in sleep duration over a four-year exposure period was associated with an increased risk of developing MetS in analyses adjusted for age, sex, sleep duration at baseline, sex, marital status, monthly income per family member, education level, smoking status, drinking status, physical activity, body mass index, snoring status and resting heart rate. A set of sensitivity analyses further confirmed these findings.

After 3.4 years of follow-up, the overall incidence of MetS in this study was 40.0%, which was significantly higher than a previously published report about midlife adults comprising a sub-cohort of ARIRANG and Quebec Family Study patients^6^,^7^,^8^. A recent meta-analysis and several prospective studies have suggested that short rather than long sleep duration is significantly associated with increased risk of MetS^2^,^6^,^7^,^8^. Our study results were quite similar to the above-published study. However, some studies have shown that longer sleep duration may also be a risk factor in participants^9^,^10^. Prior evidence connecting sleep duration to cardiometabolic risk is varied^17^, with a reported U-shaped relationship between sleep duration and MetS^18^, type 2 diabetes^19^,^20^, and mortality^13^,^21^. Various factors may contribute to this difference, such as geographic and ethnic variations, varying clinical definitions of MetS, limited confounding factors, and major diseases affecting sleep duration. Thus, in our study, a greater quantity of important influencing factors has been analyzed than in previous research, including RHR and snoring status. First, taking into consideration prior evidence showing RHR is an independent risk factor for existing MetS and a powerful predictor for future incidence of MetS^22^,^23^,^24^,^25^, we adjusted for RHR in our full model. Second, poor sleep efficiency, often relating to snoring status, has a significant correlation with over-activity of the sympathetic nervous system, which could result in insulin resistance^26^ and increased blood pressure^27^. In light of this, snoring was considered as a confounding factor in our assessment of the relative risks for MetS. Moreover, age, sex, socioeconomic status may contribute to the association between the altered sleep duration and incident MetS, as advanced age is associated with changes in sleep architecture with increased difficulties in sleep initiation and maintenance^28^. And, sleep problems are particularly common in people with anxiety, depression, bipolar disorder, and poor socioeconomic status. Compared to men, women are more likely to develop depression and sleep problems. Therefore, we adjusted for age, sex, income level and education level in the full model. Finally, subjects with stroke, myocardial infarction, and cancer may experience sleep deprivation, which could lead to biased results. Therefore, we further confirmed our finding that short sleep duration increases the risk of MetS incidence by repeating the same analysis and excluding individuals with stroke, myocardial infarction and cancer, respectively.

An inherent limitation of previous studies has been the reliance on a single time point by which to assess sleep duration, which may have occurred several decades prior to the event, and is therefore likely to yield biased estimates of the association. Moreover, there has been no consideration of how sleep duration varies within individuals over time, and the subsequent impact this could have on changes in sleep duration and future risk of MetS. As the current study appears to be the first to demonstrate an association between decreased sleep duration and incidence of MetS in a large cohort study from China, our findings thus provide the first evidence that change in sleep duration is not only strongly associated with future risk of MetS, it is also likely to be a more accurate indicator of the true magnitude of risk as compared with a single measure of sleep duration self-reported several years before the onset of MetS.

It is an interesting and new observation that a decrease in sleep duration over a 4-year period is more deleterious than persistent short sleep. This may be related to the shortened sleep duration (≤5.5 h) among sleepers with a significant decrease in sleep duration. There are a number of biological mechanisms through which reduced sleep duration may lead to MetS. Experiments have demonstrated short-term sleep deprivation among healthy subjects results in adverse physiological changes, including decreased glucose tolerance and increased insulin resistance, sympathetic tone and blood pressure^29^.

The strengths of our study include a prospective cohort design, large sample size, the Asian ethnicity of our participants, and a broad spectrum of potential confounding parameters. However, there are several potential limitations of our study. First, we only collected information on sleep duration by self-reported questionnaires, without 24-h polysomnography information. However, self-reported measures showed good agreement when compared to quantitative sleep assessment with monitoring^30^,^31^. Second, our study did not exclude participants with obstructive sleep apnea syndrome, and there is some evidence that sleep apnea is associated with increased risk of MetS^32^. Furthermore, we did not collect sufficient information on the pre- or post-menopause status of women, which appears to be an important determinant of MetS risk in women^8^. Previous studies have reported gender-specific differences in sleep patterns may be influenced by differences in social or household roles, or sex hormones^33^. Finally, most of the participants from the Kailuan coal mine were male; thus, the sex distribution of participants was unbalanced and cannot be viewed as a representative sample of the general Chinese population.

In conclusion, our findings demonstrate an association between reduced sleep duration and increased future risk of MetS. This study also highlights the need to take into consideration change of sleep duration when estimating risk, rather than relying on a single measure of exposure that often precedes the outcome by several decades. Our results should encourage and support individuals to maintain or adopt a 7-h sleep duration each night, as this could have significant beneficial effects in stemming the growing prevalence of MetS.

## Methods

### Ethics Statement

In compliance with the Declaration of Helsinki, the protocol for this study was approved by the Ethics Committee of Kailuan General Hospital and all participants provided informed written consent with signatures^34^,^35^.

### Study Design and Participants

**Figure 1:**
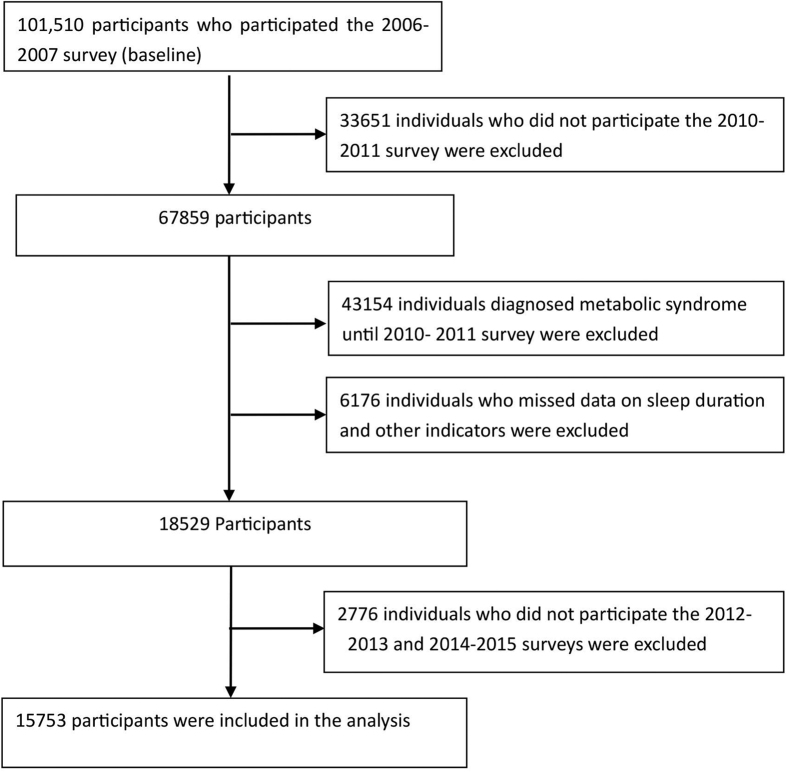
Selection of Kailuan study participants for analysis.

The Kailuan study was a prospective cohort study designed to investigate the association of risk factors and chronic disease. The Kailuan community, located at the center of the Kailuan Coal Industry in Hebei Province, China, has approximately 7.2 million inhabitants with 11 hospitals responsible for their healthcare. From June 2006 to October 2007, a total of 155,418 employees (including retired individuals) in the community were invited to participate and 65.31% of them agreed to be participants. A total of 101,510 participants (81,110 men and 20,400 women, aged 18–98 years old) were recruited into the Kailuan study. During baseline analysis, a total of 85,757 participants were excluded from the recruited population, including 33,651 participants lacking face-to-face follow-up data during 2010–2011 survey, 43,154 participants diagnosed with MetS prior to the 2010–2011 survey, and 6,176 participants lacking complete data regarding sleep duration and other indicators. In addition, 2,776 individuals who did not participate in the 2012–2013 and 2014–2015 surveys were excluded. The remaining 15,753 participants were included in the final analysis (Fig. 1). Follow-up evaluations included biennial measurement of laboratory parameters and recording of adverse events. All physicians and nurses had rigorous, unified training before conducting this study.

### Assessment of Potential Covariates

All participants underwent a clinical examination and standardized interview. Physical activity was evaluated based on individual responses to questions regarding the types and frequencies of physical activity at work and during leisure time. Physical activity was classified as “≥4 times per week and ≥20 min at a time”, “<80 min per week”, or “none”. Smoking and drinking statuses were classified as “never”, “former”, or “current” according to self-reported information. Monthly income per family member (at baseline) was categorized as “<¥600”, “¥600–799”, “¥800–999” and “≥¥1,000”.

Anthropomorphic parameters such as height, weight, and waist circumference were measured. Body mass index (BMI) was calculated as weight/height (kg/m^2^). Systolic blood pressure (SBP) and diastolic blood pressure (DBP) were measured thrice in the seated position using a mercury sphygmomanometer, and the average of three readings was used for analyses.

Blood samples were collected from the antecubital vein after an overnight fast. Venous blood was obtained for determination of routine chemistry, including fasting blood glucose (FBG), high-density lipoprotein-cholesterol (HDL-C), total cholesterol (TC), and triglycerides (TG). Resting heart rate (RHR) was measured and calculated from electrocardiogram recordings after subjects acclimated to the hospital setting for ≥30 min and were in the supine position for ≥5 min.

### Assessment of Sleep Duration

Sleep duration was elicited by the question “How many hours of sleep have you gotten on an average night in the preceding 3 months?” Response categories were ≤5, 6, 7, 8, and ≥9 h. Sleep duration in 2006–2007 and 2010–2011 was used to determine changes in sleep duration over the two exposure periods. To calculate change, baseline sleep duration (2006–2007) was subtracted from the sleep duration reported at follow-up (2010–2011). Decreased sleep was defined as a decrease of ≥2 h in sleep duration; whereas, increased sleep was defined as an increase of ≥2 h in sleep duration. Changes in sleep duration with the range of 0–1 h between 2006 and 2010 surveys was not considered to be different and classified as ‘no change in sleep duration’. For these stable sleepers, average sleep duration was calculated and categorized into five levels: ≤5.5, 6.0–6.5, 7.0, 7.5–8.0 and ≥8.5 h.

In addition, participants were asked, “Do you generally snore when you sleep?” Response alternatives were “yes” and “no.”

### Follow-Up and Diagnosis of Metabolic Syndrome (MetS)

Participants were followed up by face-to-face interviews at every 2-year routine medical examination until December 31, 2015, or to the event of interest or death. Follow-ups were performed by trained physicians who were blinded to baseline data. MetS was diagnosed when a participant had three or more of the following components: 1) Waist circumference ≥90 cm for men or ≥80 cm for women; 2) TG ≥1.7 mmol/l; 3) HDL-C <1.03 mmol/l for men or <1.30 mmol/l for women; 4) SBP/DBP ≥130/85 mmHg or current use of antihypertensive medications; 5) FBG ≥5.6 mmol/l, previous diagnosis of type 2 diabetes, or current use of oral hypoglycemic agents or insulin^36^.

### Statistical analysis

Continuous variables were expressed as means ± standard deviations; whereas, categorical variables were expressed as percentages. We compared parameters according to each sleep duration group. One-way analysis of variance (ANOVA) was used for non-paired samples of normally distributed parameters and the Kruskal-Waillis test was applied for non-parametric variables. A Chi-squared test was applied to compare categorical variables. A multivariate analysis was performed using two models: Model 1 was adjusted for age, sex, and sleep duration at baseline; Model 2 included Model 1 parameters plus monthly income per family member, education level, marital status, smoking status, drinking status, physical activity, BMI, snoring status and RHR; We used Cox proportional hazards modeling to calculate the hazard ratio (HR) and 95% confidence interval (CI) of MetS, using the group with persistent 7-h sleep duration as a reference category. Person-years were calculated from the date of the 2010 survey was conducted to the date when MetS was detected (depending on the analysis in question), date of death or date of participating in the last interview in this analysis, whichever came first. Further, as individuals with major fatal diseases could impact our assessment of sleep duration and future MetS risk, we conducted three sensitivity analyses to test the robustness of our findings by repeating our aforementioned analysis and excluding individuals with stroke, myocardial infarction and cancer, respectively. Statistical analysis was performed using SAS 9.3 statistical software (SAS Institute, Cary, NC).

## Additional Information

**How to cite this article**: Song, Q. *et al*. Changes in sleep duration and risk of metabolic syndrome: the Kailuan prospective study. *Sci. Rep.* **6**, 36861; doi: 10.1038/srep36861 (2016).

**Publisher’s note:** Springer Nature remains neutral with regard to jurisdictional claims in published maps and institutional affiliations.

## Figures and Tables

**Table 1:** Baseline characteristics of study population by incident metabolic syndrome.

	Incident metabolic syndrome		
	no	yes	*P-value*
No.	9451	6302	
Male, n (%)	8016 (84.82)	5022 (79.69)	<0.001
Age (years)	47.68 ± 12.00	46.80 ± 11.50	<0.001
Marital status (married), %	9011 (95.34)	6008 (95.33)	0.98
High-school graduate, %	2058 (21.78)	1363 (21.63)	0.83
Income≥800¥, %	1240 (13.12)	833 (13.22)	0.86
Physical activity >4 times/week, %	1214 (12.85)	766 (12.15)	0.20
Current smoker,%	3781 (40.01)	2575(40.86)	0.28
Current alcohol drinker, %	3966 (41.96)	2673 (42.42)	0.57
Body mass index (kg/m^2^)	22.50 ± 2.66	23.74 ± 2.74	<0.001
Waist circumference (cm)	80.24 ± 8.54	82.48 ± 8.72	<0.001
Systolic blood pressure (mmHg)	123.30 ± 18.25	125.14 ± 18.96	<0.001
Diastolic blood pressure (mmHg)	79.71 ± 10.66	81.11 ± 11.04	<0.001
Fasting blood glucose (mmol/L)	5.08 ± 1.12	5.18 ± 1.28	<0.001
Triglycerides (mmol/L)	1.12 ± 0.79	1.28 ± 0.90	<0.001
High density lipoprotein (mmol/L)	1.63 ± 0.38	1.56 ± 0.37	<0.001
Resting heart rate (beats/min)	72.76 ± 9.81	73.40 ± 9.79	<0.001
History of stroke,%	78 (0.83)	67 (1.06)	0.40
History of myocardial infarction,%	69 (0.73)	51 (0.81)	0.58
History of cancer,%	27 (0.29)	22 (0.35)	0.48
Sleep duration at baseline (h)	7.28 ± 1.08	7.24 ± 1.07	<0.001
Snoring status (snored),%	871 (9.22)	623 (9.89)	<0.001

**Table 2:** Baseline characteristics according to change of sleep duration.

	Sleep duration group							
	≤5.5 h	6.0–6.5 h	7 h	7.5–8 h	≥8.5 h	≥2 h decrease	≥2 h increase	*P-value*
No. of participants	707	2360	598	7824	172	2503	1589	
Male, n (%)	616 (87.13)	2058 (87.20)	524 (87.63)	6183 (79.03)	121 (70.35)	2086 (83.34)	1450 (91.25)	<0.001
Age (years)	50.39 ± 11.86	47.45 ± 11.34	43.22 ± 10.88	46.55 ± 11.83	41.59 ± 13.01	47.07 ± 11.30	52.18 ± 11.68	<0.001
Marital status (married), %	664 (93.92)	2232 (94.58)	558 (93.31)	7502 (95.88)	156 (90.70)	2414 (96.44)	1493 (93.96)	<0.001
High-school graduate, %	151 (21.36)	782 (33.14)	245 (40.97)	1418 (18.12)	71 (41.28)	468 (18.70)	286 (18.00)	<0.001
Income≥800¥, %	151 (21.36)	488 (20.68)	150 (25.08)	764 (9.76)	32 (18.60)	274 (10.95)	214 (13.47)	<0.001
Physical activity >4 times/week, %	162 (22.91)	410 (17.37)	112 (18.73)	676 (8.64)	26 (15.12)	223 (8.91)	371 (23.25)	<0.001
Current smoker,%	426 (60.25)	1433 (60.72)	351 (58.70)	2286 (29.22)	65 (37.79)	864 (34.52)	931 (58.59)	<0.001
Current alcohol drinker, %	454 (64.21)	1527 (64.70)	410 (68.56)	2360 (30.16)	65 (37.79)	879 (35.12)	944 (59.41)	<0.001
Body mass index (kg/m^2^)	22.60 ± 2.54	23.00 ± 2.62	22.84 ± 2.53	23.06 ± 2.83	22.45 ± 2.48	22.92 ± 2.76	23.13 ± 2.81	<0.001
Waist circumference (cm)	80.31 ± 8.11	80.68 ± 8.12	79.60 ± 7.85	81.22 ± 8.80	78.64 ± 8.33	81.77 ± 8.75	81.63 ± 9.22	<0.001
Systolic blood pressure (mmHg)	122.89 ± 17.44	122.76 ± 17.57	121.05 ± 16.70	124.56 ± 19.06	117.20 ± 17.14	123.74 ± 18.60	126.21 ± 18.26	<0.001
Diastolic blood pressure (mmHg)	79.25 ± 10.23	79.44 ± 10.14	78.93 ± 10.27	80.64 ± 11.18	77.64 ± 9.70	80.26 ± 10.76	80.94 ± 10.61	<0.001
Fasting blood glucose (mmol/L)	5.07 ± 1.15	5.13 ± 1.23	5.12 ± 1.09	5.12 ± 1.16	5.21 ± 1.42	5.10 ± 1.23	5.18 ± 1.22	<0.001
Triglycerides (mmol/L)	1.10 ± 0.70	1.23 ± 1.03	1.30 ± 1.04	1.18 ± 0.78	1.25 ± 1.20	1.14 ± 0.74	1.21 ± 0.86	<0.001
High density lipoprotein (mmol/L)	1.63 ± 0.42	1.60 ± 0.37	1.59 ± 0.40	1.60 ± 0.37	1.56 ± 0.34	1.59 ± 0.38	1.64 ± 0.39	<0.001
Resting heart rate (beats/min)	72.76 ± 10.08	72.56 ± 10.00	73.39 ± 10.50	73.30 ± 9.70	74.85 ± 10.40	72.71 ± 9.70	72.47 ± 9.74	<0.001
History of stroke,%	11 (1.56)	27 (1.14)	2 (0.33)	50 (0.64)	3 (1.74)	17 (0.68)	35 (2.20)	<0.001
History of myocardial infarction,%	6 (0.85)	23 (0.97)	6 (1.00)	33 (0.42)	2 (1.16)	12 (0.48)	38 (2.39)	<0.001
History of cancer,%	6 (0.85)	8 (0.34)	1 (0.17)	18 (0.23)	0 (0.00)	9 (0.36)	7 (0.44)	<0.001
Sleep duration at baseline (h)	5.19 ± 0.71	6.32 ± 0.49	7.00 ± 0.00	7.86 ± 0.37	8.58 ± 0.63	7.93 ± 0.63	5.57 ± 0.93	<0.001
Snoring status (snored),%	118 (16.69)	332 (14.07)	85 (14.21)	464 (5.93)	12 (6.98)	182 (7.27)	301 (18.94)	<0.001

**Table 3:** Association between average sleep duration and change in sleep duration and subsequent incident metabolic syndrome.

Sleep duration	Events (n,%)	N = (15753)	HR (95%CI)	
			Model1*	Model2‡
**Average sleep duration**				
≤5.5 h	305 (43.14)	707	1.14 (0.94–1.38)	1.22 (1.01–1.50)
6.0–6.5 h	978 (41.44)	2360	1.14 ((0.99–1.33)	1.14 (0.98–1.33)
7 h	225 (37.63)	598	reference	reference
7.5–8 h	3052 (39.01)	7824	1.98 (0.94–1.24)	1.12 (0.97–1.30)
≥8.5 h	70 (40.70)	172	1.20 (0.91–1.59)	1.24 (0.93–1.66)
**Change in sleep duration**				
≥2 h decrease in sleep	1045 (41.75)	2503	1.18 (1.02–1.38)	1.23 (1.05–1.44)
≥2 h increase in sleep	627 (39.46)	1589	1.07 (0.90–1.26)	1.07 (0.90–1.27)

Model 1 *Adjusted for age (years), sex, and sleep duration at baseline.

Model 2 ^‡^Adjusted for as model 1 plus marital status, monthly income per family member, education level, smoking status, drinking status, physical activity, BMI, snoring status and resting heart rate.

**Table 4:** Association between average sleep duration and change in sleep duration, and subsequent incident metabolic syndrome after excluding individuals with major diseases.

Sleep duration	HR (95%CI)‡	Sensitivity analysis2§	Sensitivity analysis3Ψ
	Sensitivity analysis1φ		
**Average sleep duration**			
≤5.5 h	1.23 (1.00–1.50)	1.23 (1.00–1.50)	1.22 (1.00–1.49)
6.0–6.5 h	1.14 (0.98–1.33)	1.14 (0.98–1.33)	1.14 (0.98–1.33)
7 h	Reference	Reference	Reference
7.5–8 h	1.12 (0.0.97–1.30)	1.12 (0.97–1.30)	1.12 (0.97–1.30)
≥8.5 h	1.25 (0.93–1.68)	1.26 (0.94–1.69)	1.24 (0.93–1.66)
**Change in sleep duration**			
≥2 h decrease in sleep	1.23 (1.05–1.44)	1.22 (1.04–1.43)	1.22 (1.04–1.43)
≥2 h increase in sleep	1.08 (0.90–1.28)	1.08 (0.91–1.29)	1.07 (0.90–1.28)

^‡^Model 2: Adjusted for age (years), sex, sleep duration at baseline, marital status, monthly income per family member, education level, smoking status, drinking status, physical activity, body mass index, snoring status and resting heart rate.

^φ^Adjusted for model 3 and further excluded individuals with history of stroke.

^§^Adjusted for model 3 and further excluded individuals with history of myocardial infarction.

^Ψ^Adjusted for model 3 and further excluded individuals with history of cancer.
